# High-Risk Acute Coronary Syndrome in a Patient with Coronary Subclavian Steal Syndrome Secondary to Critical Subclavian Artery Stenosis

**DOI:** 10.1155/2014/175235

**Published:** 2014-08-04

**Authors:** Zaher Fanari, Niksad Abraham, Sumaya Hammami, Wasif A. Qureshi

**Affiliations:** ^1^Section of Cardiology, Christiana Care Health System, Newark, DE 19718, USA; ^2^School of Public Health, Saint Louis University, St Louis, MO, USA

## Abstract

Patients with multivessel coronary artery disease are more likely to have extensive atherosclerosis that involves other major arteries. Critical subclavian artery (SCA) stenosis can result in coronary subclavian steal syndrome that may present as recurrent ischemia and even myocardial infarction in patients with coronary artery bypass graft (CABG). In patients with concomitant severe native coronary disease, occluded saphenous venous grafts (SVG) to other arteries, percutaneous intervention on critical subclavian artery (SCA) stenosis that will compromise the blood flow to left internal mammary graft (LIMA) and left anterior descending (LAD) artery will be a high-risk procedure and may be associated with cardiogenic shock, especially in patients with preexisting ischemic cardiomyopathy. The use of percutaneous left ventricular (LV) assist device like Impella will offer better hemodynamic support and coronary perfusion and therefore results in decreased myocardial damage, maximized residual cardiac function, and lower incidence of cardiogenic shock.

## 1. Introduction

Patients with multivessel coronary artery disease are more likely to have extensive atherosclerosis that involves other major arteries. Critical subclavian artery (SCA) stenosis is known to be associated with “steal syndrome” that usually presents with neurological symptoms. In patients with CAD after CABG and especially if the patient has extensive CAD, limited collaterals, and/or occluded grafts, SCA stenosis may presents as coronary subclavian steal syndrome which will present with recurrent ischemia [5, 7] and if this stenosis becomes critical this may results in a myocardial infarction [1].

Percutaneous coronary interventions (PCIs) in patients with multivessel disease and low ejection fraction (EF) are considered high-risk interventions that can easily result in complications including coronary hypoperfusion, heart failure, and hemodynamic collapse [2, 3]. The use of prophylactic support device helps in unloading the LV and improves coronary perfusion. A percutaneous left ventricular (LV) assist device significantly reduces the risk of such procedures and helps improving patient outcomes [4].

We report the first case of Impella left ventricular (LV) assist device to provide successful PCI support in critical subclavian artery (SCA) stenosis in a patient with severe native obstructive CAD, occluded saphenous venous grafts (SVG), and one patent left internal mammary graft (LIMA) to LAD that is jeopardized by the SCA stenosis with the support of Impella left ventricular (LV) assist device. This is the first reported case where Impella is used to provide support while performing intervention on critical SCA stenosis.

## 2. Case Report

**Figure 1:**
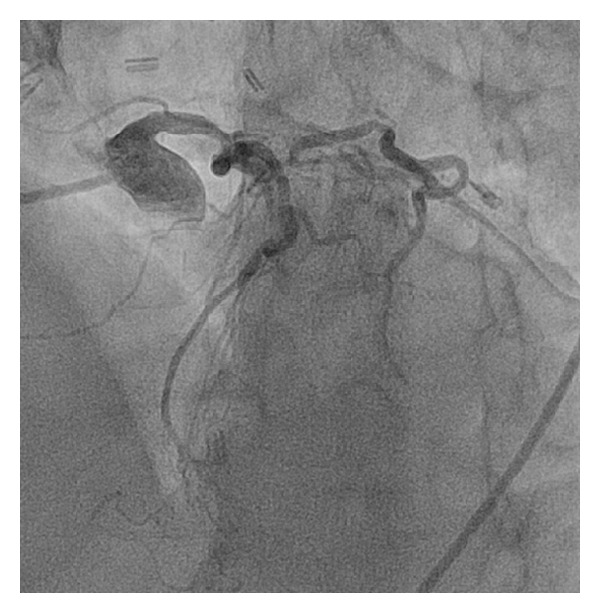
Angiography of left coronary system shows extensive native disease.

**Figure 2:**
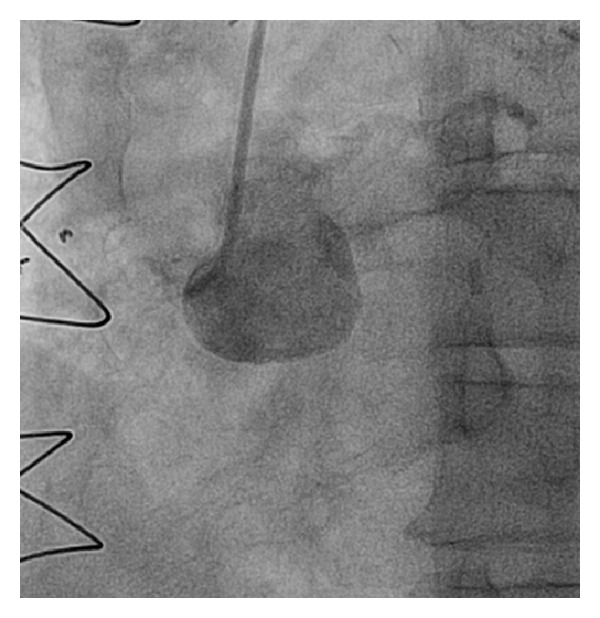
Chronically occluded RCA.

**Figure 3:**
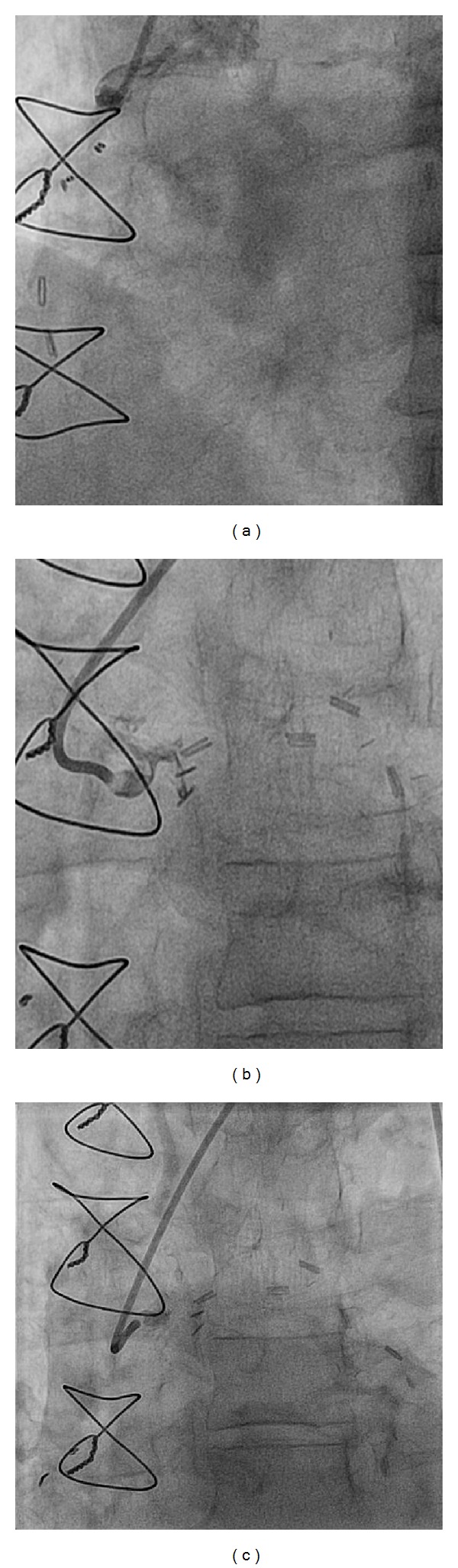
(a) Occluded SVG to RCA. (b) Occluded SVG to circumflex. (c) Occluded SVG to obtuse marginal 1.

The patient is a 67-year-old Caucasian male with hypertension, hyperlipidemia, long history of smoking, Type 2 diabetes mellitus, and CAD s/p four-vessel bypass 8 years ago (LIMA to LAD, SVG to left circumflex, obtuse marginal, and RCA) who presented with chest pain, ST depression in the anterior lead of V2–V6, and elevated troponin. A diagnosis of NSTEMI was made and the patient was taken to the catheterization lab. A cardiac catheterization revealed severe native left and right system disease (90% stenosis in left main, 90% in ostial left circumflex, and complete occlusion of the midportion of LAD (Figure 1) and ostium of RCA (Figure 2). His three venous bypass grafts were seen to be occluded (Figures 3(a), 3(b), and 3(c)) and his LIMA was patent. The left subclavian artery had a calcified 99% ostial and proximal stenosis with 80 mm Hg gradient. His LVEF was seen to be at 35–40%. As he had only one patent bypass graft, NSTEMI, ischemic cardiomyopathy, and limited collaterals, this was considered as very high risk and the decision was made to perform this intervention under Impella support.

## 3. Procedure Details

**Figure 4:**
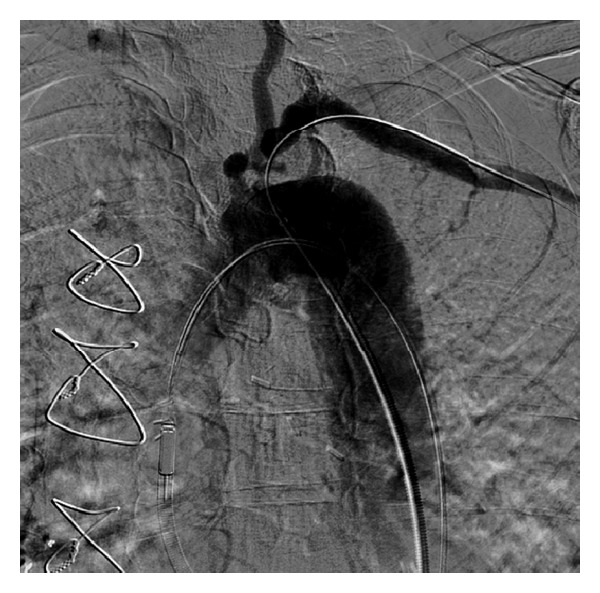
Critical ostial subclavian artery stenosis.

**Figure 5:**
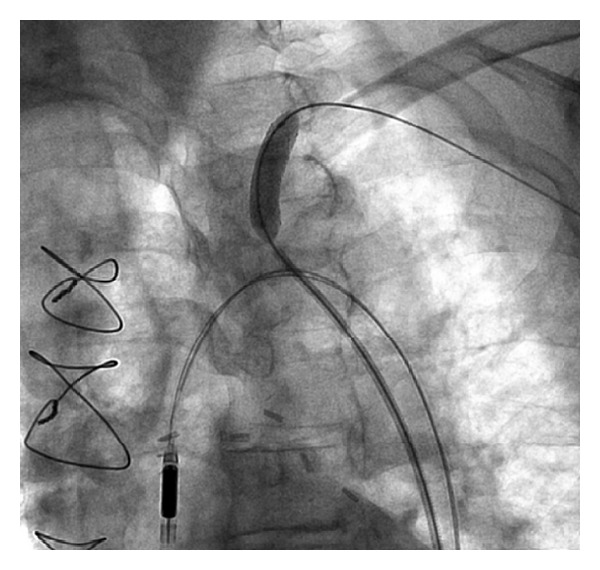
A stent deployment in the subclavian artery with the support of Impella.

**Figure 6:**
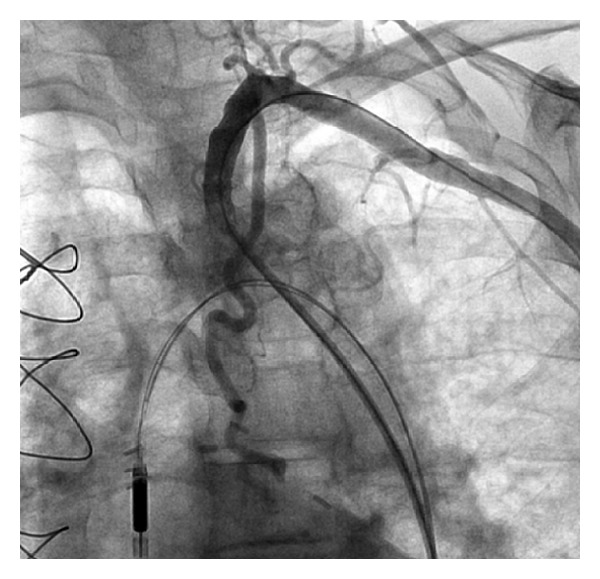
TIMI 3 flow with no residual stenosis after stenting of subclavian artery.

Bilateral CFA angiograms were performed. He had tortuosity and mild atherosclerotic vascular disease in the common and external iliac arteries bilaterally with the left more conducive to the Impella. Thus, it was decided to place the Impella via the left CFA. Preclose technique was applied using two six-French perclose devices and then using a stiff Amplatz wire and after sequential dilatations with 8 and 12 French dilators a 14-French 30 cm sheath was then placed. The Impella 2.5 percutaneous assist device was then placed across the aortic valve and was seen to be providing excellent support. A head hunter 1 catheter was then used with an angled glide wire to successfully cross the lesion in the left subclavian (Figure 4). There was a pressure gradient of about 80 mm Hg. An Amplatz super stiff wire was then advanced and placed distally in the left axillary artery. The head hunter catheter and the short 6-French sheath was then exchanged for a 90 cm six-French destination sheath which was then used to engage the left subclavian. A 4.0 × 20 mm balloon was then used to predilate the lesion. Then an 8.0 × 27 mm VISIPRO stent was deployed in the ostium of the left subclavian at 10 atmospheres for 15 seconds with 0% residual stenosis, TIMI 3 flow, and no dissection (Figure 5). The LIMA graft was seen to be preserved with excellent TIMI 3 flow (Figure 6).

The Impella was then weaned and then removed. The 14-French sheath was then removed and closure was done in the left CFA over the wire. The wire was then removed and manual pressure was held for 10 minutes with complete hemostasis. The destination sheath in the right groin was removed and angioseal closure device was deployed. There were no complications. On peripheral testing there was no gradient between the subclavian arteries. On follow-up, the patient had no recurrence in angina or shortness of breath.

## 4. Discussion

Coronary subclavian steal syndrome is a rare phenomenon that is reported to occur in 0.4% to 1.1% of CABG patients results when a critical atherosclerotic change causes a stenosis of the subclavian artery, which in turn causes a limited coronary flow in patients with LIMA bypass grafting to the left coronary [5]. Proximal stenosis of the subclavian artery can cause reductions of flow within the left IMA graft and result in myocardial ischemia with or without reversal of flow within the vertebral artery [6]. If the stenosis becomes critical or an acute occlusion occurs in the subclavian artery, this may result in myocardial infarction [1]. Many cases were reported about these phenomenon and stenting of subclavian artery results in improvement of ischemia [1, 5–8]. In all of these cases the coronary anatomy through CABG was reported to be mainly unaffected and no support left ventricle devises were needed.

In patients with extensive multivessel coronary disease especially when associated with LV dysfunction and/or occluded SVG grafts, PCI is considered a very high risk procedure and in many cases providers abstain from performing the procedure because of this high risk [4]. This high risk results from hemodynamic effects of the ischemia that may be induced by obstructive balloon dilation, and possible atheroembolism into the LIMA. This ischemia may induce hemodynamic compromise especially when there is poor collateral flow. LV assist devices can provide excellent cardiac support during the procedure and help manage potential emergencies like cardiogenic shock in high-risk patients [2, 9].

Although that the intra-aortic balloon pump is the most commonly used support device, it may not provide enough hemodynamic support in patients with extremely low EF or who might be at much higher risk for cardiogenic shock [4]. The use of Impella Recover 2.5 on the other hand offers many advantages. By unloading the myocardium, it offers better hemodynamic support and coronary perfusion and therefore results in decreased myocardial damage, maximized residual cardiac function, and lower incidence of cardiogenic shock [10]. Furthermore; it is easy to use device and it can be placed through peripheral artery and can be kept safely for further support up to 5 days [11]. Also data that support the safety of both short and long-term use of impella are available through multiple trials [3, 12]. The use of such a device is usually preceded with an echocardiography to exclude the presence of preprocedure LV thrombus, as these high-risk patients who qualify for the use of this device are more prone to have an intracardiac thrombus [13]. This device despite its safety is still associated with some complications including hemolysis, vascular compromise, aortic regurgitation, infection, bleeding, hematoma, and limb ischemia [2, 11, 12].

The potential benefit of Impella over IABP comes from PROSPECT II trial [14, 15] that randomized 452 symptomatic patients with complex 3-vessel disease or unprotected left main coronary artery disease and severely depressed left ventricular function to intra-aortic balloon pump (IABP) or Impella 2.5 support during nonemergent high-risk percutaneous coronary intervention. Impella 2.5 was shown to provide superior hemodynamic support in comparison with IABP, with maximal decrease in cardiac power output from baseline of −0.04 ± 0.24 W in comparison with −0.14 ± 0.27 W for IABP (*P* = 0.001). However this superior hemodynamic support did not turn to a significant statistical difference in the primary end point of major adverse events MAE at 30 days (35.1% for Impella 2.5 versus 40.1% for IABP, *P* = 0.227) [14]. A subsequent analysis of the data at 90 days showed that when compared to IABP, Impella use was associated with lower incidence in both MAE (37% versus 49%, *P* = 0.014) and major adverse cardiac and cerebral events (MACCE) (22% versus 31%; *P* = 0.034) [15].

Our patient is presenting in unique clinical, hemodynamic, and anatomical challenges. The patient presented with NSTEMI and LV dysfunction. He had severe extensive native coronary artery disease with completely occluded LAD and RCA and high degree stenosis in both the left main and circumflex arteries. Furthermore his CABG, which included LIMA and 3 SVG, was compromised by 3 occluded SVG. The only patent graft, which is the LIMA graft to LAD, had a compromised flow by a critical stenosis in the subclavian artery. The only way to prevent a future STEMI and potential death in this gentleman can be offered by doing percutaneous intervention on the major artery that constitutes the only blood supply to his cardiac muscle. In our case the use of Impella device enabled us to keep the patient hemodynamically stable for a sufficient amount of time so that the subclavian artery intervention was completed. Although there are several large series that reported the use of the Impella in high-risk PCI [3, 4, 11], all these cases were specifically coronary interventions and our case is the first case to report the use of prophylactically LV support device in a subclavian artery intervention.

## 5. Conclusion

The use of percutaneous left ventricular (LV) assist device like Impella in patients with coronary subclavian steal syndrome and extensive native and graft coronary artery disease will offer better hemodynamic support and coronary perfusion and therefore results in decreased myocardial damage, maximized residual cardiac function, and lower incidence of cardiogenic shock.
